# Email invitations to publish: Academically sound (such as *SAFP*) versus potentially predatory journals

**DOI:** 10.4102/safp.v66i1.5984

**Published:** 2024-09-25

**Authors:** Gina Joubert, Omololu Aluko

**Affiliations:** 1 Department of Biostatistics, Faculty of Health Sciences, University of the Free State, Bloemfontein, South Africa

**Keywords:** email, invitation, articles, publications, accredited journals

## Abstract

**Background:**

Researchers increasingly receive invitations by email to publish. We analysed email publication invitations received by staff members of the Department of Biostatistics, University of the Free State (UFS), comparing emails relating to accredited and non-accredited journals.

**Methods:**

This cross-sectional study included all publication invitations received via UFS email accounts by staff members from May 2023 to July 2023. The researchers independently completed the data form, then checked and resolved any discrepancies.

**Results:**

Of the 93 distinct emails received from 88 journals, only 15 (16%) were received from a journal appearing on the Department of Higher Education and Training (DHET) accredited journal lists. These included *South African Family Practice* (SAFP) and the *African Journal of Primary Health Care and Family Medicine* (PHCFM). Emails from non-accredited journals were significantly (*p* < 0.01) less likely to refer to a journal with a health sciences-related title (37% vs. 86%), indicate the publisher (36% vs. 93%), provide a link to the journal website (59% vs. 100%), state a full physical address (24% vs. 80%), refer to author instructions (21% vs. 47%) or request the recipient to share the email with colleagues (5% vs. 47%). Emails from non-accredited journals were significantly (*p* < 0.01) more likely to contain grammatical errors (63% vs. 0%) and flattering remarks regarding the recipient or his or her research work (49% vs. 0%), and to indicate the journal’s International Standard Serial Number (ISSN) (67% vs. 13%).

**Conclusion:**

Clear differences were found between email invitations from accredited versus non-accredited journals.

**Contribution:**

The findings provide insight into warning signals in email publication invitations.

## Introduction

Researchers are increasingly approached by journals via email to publish their research and are targeted, especially once they have published and their contact email is available online.^1^ Amidst this deluge,^2^ authors ‘have a responsibility to evaluate the integrity, history, practices, and reputation of the journals to which they submit manuscripts’.^3^ In South Africa, the Department of Higher Education and Training (DHET) annually releases a list of accredited journals consisting of international lists, such as Web of Science and Scopus, as well as selected South African journals not appearing on the international lists.^4^ There is, on the other hand, a large number of suspect journals publishing thousands of manuscripts per year, increasingly in special issues. For example, *International Journal of Environmental Research and Public Health* (IJERPH) published approximately 17 000 manuscripts in 2022,^5^ more than 1400 articles per month. One of the aspects on which advice has been given to judge a journal’s integrity is the type of email invitation sent.^6^ Poor grammar, flattering language and the promise of quick acceptance are some of the items identified as indicative of so-called predatory journals. Promised quick acceptance can appeal to inexperienced researchers as journal turnaround times can be lengthy.^7^

The aim of this study was to analyse email publication invitations received by staff members of the Department of Biostatistics, University of Free State (UFS), comparing emails received from accredited and non-accredited journals.

## Methods

This cross-sectional study included all publication invitations received via UFS email accounts by staff members (five full-time staff members plus one affiliate associate professor) of the Department of Biostatistics, UFS, over the 3-month period May 2023 – July 2023. Staff members were approached to participate by the second author. Staff members who gave written informed consent provided their publication email invitations to the researchers electronically. A data form was compiled based on previous studies^1,8,9^ and the researchers’ experience. The researchers independently completed the data form on REDCap (REDCap Consortium; Fort Lauderdale, Florida, United States [US]) for each email received. Each researcher checked whether the journal appeared on the DHET list of accredited journals available on the university library website. Data were exported to an Excel spreadsheet (version 365) (Microsoft Corporation; Redmond, Washington, US). All discrepancies between the researchers’ entries were flagged. Each researcher checked through their entries regarding these discrepancies, and after two rounds of such checking, the researchers discussed and resolved the remaining discrepancies.

A pilot study involved the two researchers independently completing data for 15 emails (the first five email publication invitations of the three staff members who received such invitations during the study period). Subsequently, a few items’ options were changed from being mutually exclusive to inclusive. The pilot study cases were included in the main study.

Results were summarised by frequencies and percentages (categorical variables) and medians and ranges (numerical variables). Chi-squared or Fisher’s exact tests were used for subgroup comparisons of categorical variables. The analysis was performed by the first author, using SAS version 9.4 (SAS Institute Inc.; Cary, North Carolina, US).

### Ethical considerations

Ethical clearance to conduct this study was obtained from the University of the Free State Health Sciences Research Ethics Committee (No. UFS-HSD2023/0801/2811) and permission was obtained from UFS authorities. No staff members were identified by any information.

## Results

Three of the staff members received a total of 129 publication invitations via their UFS email accounts during the study period. Three staff members received no such invitations. Of the 129 publication invitations, 15 (11.6%) were from a publisher, with no specific journal(s) mentioned, and 2 (1.6%) from publishers requesting submission of a book. The majority of the invitations (*n* = 112; 86.8%) were regarding specific journals, which were the focus of this analysis. Duplicate emails with the same content sent to different researchers or to the same researcher at different times were excluded from further analysis. Ninety-three distinct (i.e., differing in content) emails were received from 88 journals. Two of these journals were related to biostatistics. Fifteen of the 93 emails (16%) were from a journal listed on the accredited journal lists of the DHET. *South African Family Practice* (SAFP) and the *African Journal of Primary Health Care and Family Medicine* (PHCFM) were two of these journals.

Table 1 summarises the characteristics of the emails related to journals on the accredited journal lists and those not accredited. Emails regarding non-accredited journals were significantly (*p* < 0.01) less likely than those from accredited journals to refer to a journal with a title indicating a specific health sciences field (37% vs. 86%), indicate the name of the publisher (36% vs. 93%), provide a link to the journal website (59% vs. 100%), state a full physical address (24% vs. 80%), refer to author instructions (21% vs. 47%) or request the recipient to share the email with colleagues (5% vs. 47%).

**TABLE 1:** Characteristics of email invitations for publication in accredited versus non-accredited journals.

Items	Journal status¶							
	Accredited (*n* = 15)				Non-accredited (*n* = 78)			
	*n*	%	Median	Range	*n*	%	Median	Range
**Journal title**								
A specific health field	13	87	-	-	29	37	-	-
Health broadly	1	7	-	-	27	35	-	-
Broad, not only health	0	0	-	-	19	24	-	-
Specific field, not health	1	7	-	-	0	0	-	-
Broad, not health	0	0	-	-	3	4	-	-
**Publisher†**								
Stated in email	14	93	-	-	28	36	-	-
Could be deduced (e.g. from email address)	1	7	-	-	15	19	-	-
No indication	0	0	-	-	35	45	-	-
**Salutation**								
None	5	33	-	-	14	18	-	-
Only a greeting (Greetings; Hi)	0	0	-	-	3	4	-	-
Generic (researcher or colleague or doctor)	5	33	-	-	28	36	-	-
Name and surname	0	0	-	-	13	17	-	-
Title and surname	4	27	-	-	2	3	-	-
Title plus initial and/or name plus surname	0	0	-	-	10	13	-	-
Other (first name only, initial and surname, name and surname in strange order)	1	7	-	-	8	10	-	-
**Text of the email**								
Grammatical errors present	0	0	-	-	49	63	-	-
Spelling mistakes present	0	0	-	-	12	15	-	-
Flattering remarks made	0	0	-	-	38	49	-	-
Inappropriate remarks made	2‡	13	-	-	19	24	-	-
None of the above	13	87	-	-	13	17	-	-
Statement that this is a follow-up or reminder email	0	0	-	-	9	12	-	-
Shortage of manuscripts mentioned	0	0	-	-	5	6	-	-
Indexing mentioned	4	27	-	-	26	33	-	-
Peer review mentioned	8	53	-	-	32	41	-	-
Open access mentioned	9	60	-	-	28	36	-	-
Author instructions mentioned	7	47	-	-	16	21	-	-
ISSN stated	2	13	-	-	52	67	-	-
Journal impact factor stated	6	40	-	-	23	29	-	-
**Reference to a previous publication of the researcher**								
Yes, vague statement	3	20	-	-	2	3	-	-
Yes, specific manuscript	2	13	-	-	15	19	-	-
**Year published**								
2020	0	-	-	-	3	-	-	-
2022	0	-	-	-	5	-	-	-
2023	2	-	-	-	7	-	-	-
**Submission deadline mentioned**	7	47	-	-	31	40	-	-
Specific date indicated	6	40	-	-	27	35	-	-
Time to submission	-	-	4.5 months	5 days – 5 months	-	-	15 days	4 days –7.5 months
**Costs mentioned**	4	24	-	-	25	31	-	-
Specific costs mentioned	2	13	-	-	19	24	-	-
Cost (USD)	-	-	$2819.00§	$2640.00 – $2998.00	-	-	$30.00	$0.00 – $1100.00
Discount mentioned	4	27	-	-	11	14	-	-
**Time from submission to decision mentioned**	0	0	-	-	6	8	-	-
Time	-	-	-	-	-	-	12 days	48 h to 16 days
**Time from submission to publication mentioned**	2	13	-	-	6	8	-	-
Time	-	-	6.5 months	4–9 months	-	-	3–7 days	0–18 days
**Type of manuscript requested**								
Research article	5	36	-	-	25	32	-	-
Case report	1	7	-	-	8	10	-	-
Review	1	7	-	-	9	12	-	-
Editorial	0	0	-	-	1	1	-	-
Opinion	0	0	-	-	2	3	-	-
Mini review	0	0	-	-	3	4	-	-
Short communication	1	7	-	-	5	6	-	-
Any type	2	13	-	-	27	35	-	-
Unclear	6	40	-	-	23	30	-	-
**Topic requested**								
Some specific field or focus	9	60	-	-	11	14	-	-
Broad health	1	7	-	-	10	13	-	-
Broader than health	0	0	-	-	15	19	-	-
Broad not health	0	0	-	-	2	3	-	-
Not stated	5	33	-	-	40	51	-	-
**Submission mode**								
Only online	8	53	-	-	14	18	-	-
Only email	1	7	-	-	25	32	-	-
Online or email	0	0	-	-	22	28	-	-
Not stated	6	40	-	-	17	22	-	-
**Role requested**								
Author	15	100	-	-	77	99	-	-
Reviewer	0	0	-	-	4	5	-	-
Become editorial board member	0	0	-	-	10	13	-	-
Become editor	0	0	-	-	3	4	-	-
Not clear	0	0	-	-	1	1	-	-
Other	1	7	-	-	1	1	-	-
**Link provided to journal website**	15	100	-	-	46	59	-	-
**Full physical address provided**	12	80	-	-	19	24	-	-
Europe/United Kingdom	5	-	-	-	1	-	-	-
South Africa	5	-	-	-	2	-	-	-
US	2	-	-	-	11	-	-	-
India	0	-	-	-	4	-	-	-
US and Europe	0	-	-	-	1	-	-	-
**Name at bottom of email**								
None	7	50	-	-	32	41	-	-
Only first name	0	0	-	-	7	9	-	-
First name and surname	8	53	-	-	38	49	-	-
Other	0	0	-	-	1	1	-	-
**Request to share with colleagues**	7	47	-	-	4	5	-	-
**Unsubscribe option**	12	80	-	-	50	64	-	-

US, United States; ISSN, International standard serial number.

† , Three publishers had journals on accredited lists and journals not on accredited lists;

‡ , Included a few exclamation marks;

§ , USD, United States dollar;

¶ , Status determined according to the annual Department of Higher Education and Training (DHET) list of accredited journals.

Emails received from non-accredited journals were significantly (*p* < 0.01) more likely than those from accredited journals to contain grammatical errors (63% vs. 0%) and flattering remarks regarding the recipient or their research (49% vs. 0%), and to indicate the journal’s ISSN number (67% vs. 13%). Approximately 40% of emails mentioned a specific deadline for submission. In these cases, the median for accredited journals was 4.5 months compared to 15 days for non-accredited journals. Around a quarter of emails from non-accredited journals mentioned a specific publication cost, and the median was $30.00 (United States dollar). Only two emails regarding accredited journals mentioned specific costs and these were $2640.00 and $2998.00, respectively.

## Discussion

Although this study represents a small number of emails compared to some published studies,^2,10,11^ it investigated the unique aspect of South African accredited journals. Email publication invitations were received from accredited journals (approximately a sixth of the unsolicited emails). Researchers should thus not consider all email publication invitations as suspect. However, some non-accredited journals stated that they appeared on the Scopus or Web of Science list where this was not in fact the case. The lists are updated annually. For example, the aforementioned IJERPH was removed from the Web of Science list in 2023.^5^ Non-accredited journals frequently mentioned being indexed in, for example, Google Scholar, which does not perform quality assurance.

Given the clear differences between email invitations relating to accredited versus non-accredited journals, this study provides insight into warning signals that align with aspects mentioned in other studies.^10,12^ However, some of the aspects included in the toolkit proposed by Dadkhah et al.^13^ to detect invitations from potentially predatory journals, rarely occurred in our study (invitation to join the journal’s editorial board or reference to previous publications). Stated publication costs were notably lower (in fact, enticingly low) for articles invited by the non-accredited journals in our study compared to costs reported elsewhere for predatory journals.^11^ Increasing use of artificial intelligence to construct the emails may in future decrease the grammatical errors, inappropriate remarks and salutation issues.

All emails received indicated that the recipient should submit some sort of manuscript. The practice of offering co-authorship (for payment) for an already drafted manuscript^14^ has fortunately not yet reached our mailboxes. As most emails contained an unsubscribe option, one should be able to prevent the deluge from overwhelming one’s mailbox. By July 2024 one of the staff members received more than 60 emailed publication invitations from journals per month.

## Conclusion and recommendations

Publication invitations by email were received from accredited journals, also in the field of Family Medicine; not all email publication invitations are thus suspect. The clear differences between email invitations from accredited versus non-accredited journals provide insight into warning signals.

It is advisable to steer clear of any journals whose email publication invitations contain grammatical errors or remarks flattering the researcher or his or her research work, do not indicate the publisher and do not provide a link to the journal website. As statements that some journals make regarding indexing in, for example, Scopus or Web of Science, cannot be relied on, it is recommended that journals’ inclusion in the DHET lists are checked to ensure that one’s research is not sent to an academically questionable journal.
